# Abnormal Cognition, Sleep, EEG and Brain Metabolism in a Novel Knock-In Alzheimer Mouse, PLB1

**DOI:** 10.1371/journal.pone.0027068

**Published:** 2011-11-11

**Authors:** Bettina Platt, Benjamin Drever, David Koss, Sandra Stoppelkamp, Amar Jyoti, Andrea Plano, Aneli Utan, Georgina Merrick, Duncan Ryan, Valeria Melis, Hong Wan, Marco Mingarelli, Emanuele Porcu, Louise Scrocchi, Andy Welch, Gernot Riedel

**Affiliations:** 1 School of Medical Sciences, College of Life Sciences and Medicine, University of Aberdeen, Aberdeen, United Kingdom; 2 Translational & Molecular Medicine, Pfizer BioTherapeutics, Collegeville, Pennsylvania, United States of America; 3 Amorfix Life Sciences, Mississauga, Ontario, Canada; National Institute of Health, United States of America

## Abstract

Late-stage neuropathological hallmarks of Alzheimer's disease (AD) are β-amyloid (βA) and hyperphosphorylated tau peptides, aggregated into plaques and tangles, respectively. Corresponding phenotypes have been mimicked in existing transgenic mice, however, the translational value of aggressive over-expression has recently been questioned. As controlled gene expression may offer animal models with better predictive validity, we set out to design a transgenic mouse model that circumvents complications arising from pronuclear injection and massive over-expression, by targeted insertion of human mutated amyloid and tau transgenes, under the forebrain- and neurone-specific CaMKIIα promoter, termed PLB1_Double_. Crossing with an existing presenilin 1 line resulted in PLB1_Triple_ mice. PLB1_Triple_ mice presented with stable gene expression and age-related pathology of intra-neuronal amyloid and hyperphosphorylated tau in hippocampus and cortex from 6 months onwards. At this early stage, pre-clinical ^18^FDG PET/CT imaging revealed cortical hypometabolism with increased metabolic activity in basal forebrain and ventral midbrain. Quantitative EEG analyses yielded heightened delta power during wakefulness and REM sleep, and time in wakefulness was already reliably enhanced at 6 months of age. These anomalies were paralleled by impairments in long-term and short-term hippocampal plasticity and preceded cognitive deficits in recognition memory, spatial learning, and sleep fragmentation all emerging at ∼12 months. These data suggest that prodromal AD phenotypes can be successfully modelled in transgenic mice devoid of fibrillary plaque or tangle development. PLB1_Triple_ mice progress from a mild (MCI-like) state to a more comprehensive AD-relevant phenotype, which are accessible using translational tools such as wireless EEG and microPET/CT.

## Introduction

Genetically modified experimental mouse models have played a pivotal role in our understanding of Alzheimer's disease (AD) aetiology. Although some of these models successfully mimic disease endophenotypes (e.g. [1]), no mouse model has yet fully recapitulated human AD pathology, and translational research has not fulfilled high expectations [2]. The current gold standard termed 3xTg-AD mouse [3] was generated by pronuclear injection of two separate transgenes, i.e. human tau (P301L) and APP_SWE_, into embryos from presenilin 1 (PS146V) transgenic mice under the control of neuron-specific Thy1,2 regulatory element. This approach raises a number of questions since onset of pathology already occurs in embryonic tissue [4], transgene insertion artefacts (positional effects) cannot be identified, and the contribution of individual transgenes to emerging phenotypes are difficult to resolve. Moreover, genetic stability and thus reproducibility of data is uncertain [5], and see for example lack of plaque pathology in follow-up studies [6]. Moreover, early expression of transgenes in embryonic tissue [4] strongly suggest developmental and putative compensatory alterations, and heavy plaque load in young animals mimics late stage AD rather than mild cognitive impairment (MCI)-like, prodromal stages of the disease.

Clinical data suggest that a significant proportion of elderly people without dementia have β-amyloid (βA) deposits, while a considerable number of AD patients have severely compromised cognition but few plaques (e.g. [7], [8]). Consistently, clearance of plaques (e.g. by immunisation or secretase inhibition) did not benefit cognition [9], and it appears that soluble βA species such as intraneuronal βA oligomers bring about early pathological events and synaptic dysfunction [10], [11].

In animals, neurological as well as cognitive deficits commonly dissociate from plaque load, and anti-βA immunization of PDAPP and APP/PS1 mice successfully rescued memory deficits, but without removal of overall plaque burden [12]–[14]. Cognitive deficits thus seem to develop coincidentally prior to extracellular plaque formation [3]. Physiological and behavioural deficits detected in AD-mice expressing exclusively oligomeric βA with no fibrillar plaques [15] supports this contention.

Along the same lines, oligomeric tau species confer higher toxicity than fibrils and tangles (e.g. [16]) and such granular oligomers are present in AD patients at Braak stage 0 and intensify progressively. They can be mimicked in transgenic animal models and culture preparations (e.g. [17]), confirming the nucleation or seeding hypothesis as the key process for proteinaceous filament formation [18]. As early soluble protein species are crucial for both disease onset and toxicity, their removal would offer greatest treatment success and future research may benefit from animal models that mimic such pathologies.

To address this issue, we created an AD model based on single-copy knock-in of human, mutated APP and Tau. Endophenoptypes were assessed to characterise early AD-like events based on predominantely intracellular APP/βA and tau expression, with methods relevant for translational medicine including immunohistochemistry, electrophysiology, cognition, actimetry, electro-encephalography (EEG) and metabolic imaging using microPET.

## Results

### PLB1_Triple_ mice express mutated human APP and tau

**Figure 1:**
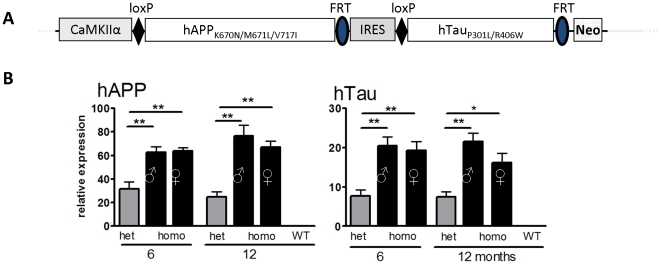
PLB1 gene construct design and expression levels. **A:** Genomic sequence of the transgene construct with the CaMKIIα promoter, human APP (hAPP), an internal ribosome entry site (IRES), followed by Tau (hTau); mutation sites are also indicated. hAPP and hTau were flanked by loxP and FRT sites, respectively. Neo: Neomycin selection cassette. **B:** The mRNA expression of hAPP (left) and hTau (right) transgenes determined in cortex was stable over time; homozygous (homo) animals showed 2–3 fold higher expression levels cf. heterozygous (het) mice. Note that hemizygous males are listed as homozygous since equal gene load was apparent cf homozygous females due to X chromosome inactivation. Relative expression levels of hAPP were ∼3 fold higher than hTau. Data (mean+SEM) were normalised to GAPDH. *: P<0.05; **: P<0.01.

PLB1 mice generated by targeted knock-in of a human APP-Tau cDNA construct (Fig. 1A) under the control of the mouse CaMKIIα promoter and crossed with PS1 mice generated a novel AD mouse line, PLB1_Triple_ (hAPP/hTau/hPS1). Litter size, overall health and attrition rates were not affected in transgenic PLB1_Triple_ mice (see Figure S2). Quantitative real-time PCR (Fig. 1B) confirmed stable forebrain expression (negligible in cerebellum) of hAPP and htau at both age groups (6 and 12 months) with low inter-subject variability. Hemizygous males and homozygous females had 2–3 fold higher hAPP and htau mRNA levels than heterozygous mice, in agreement with random X chromosome inactivation in females. Therefore, despite the presence of only one copy, gene load in hemizygous males is equivalent to homozygous females. subsequent data presented here are therefore from hemi- and homozygous PLB1_Triple_ mice based on equal gene dosage.

**Figure 2:**
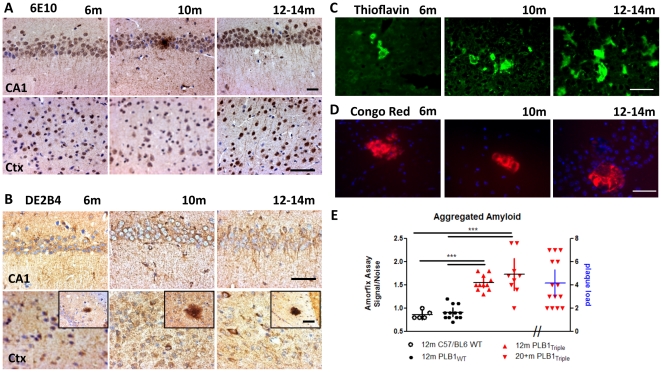
Amyloid histopathology in homozygous PLB1_Triple_ mice. **A–D:** Microphotographs from coronal sections of 6, 10, 12–14 months old animals; scalebars: 50 µm. **A–B:** Immunoreactivity towards APP/βA (6E10 and DE2B4 antibodies) was progressively seen in somata and processes of hippocampal pyramidal cells (CA1) and cortical neurones (Ctx). Occasional strong extracellular immunostaining demonstrates the formation of βA plaques, also visualised by Thioflavin-S and Congo Red fluorescence (**C&D**), confirming β-sheet protein folding and mature aggregation of extracellular deposits. Quantification of plaque load was conducted in 21 month old triples (**E**, 6E10 stain). The βA load was assessed based on the signal obtained in the Amorfix A^4^ assay for aggregated amyloid (see Methods and Text S1) and confirmed significantly enhanced levels in 12 and 20 months old PLB1_Triple_ mice compared with wild-type C57/BL6 and PLB1_WT_ lines (displayed as individual data points, mean and 95% CI, ***:P<0.001).

Immunolabelling of APP/amyloid and tau using human-specific antibodies (6E10 & DE2B4; Fig. 2) confirmed forebrain expression of both proteins at ∼5–6 months of age, with staining being absent at 3 months. Staining was preferentially detected intercellularly in hippocampus and cortex (Fig. 2A&B), and was particularly strong in somata of principal cells but progressively extended into the neuropil and dendritic layers (for PLB1_WT_ see Figure S3). Although βA levels were below the sensitivity limit of commercially available ELISAs (tested in tissue form 12 months old mice, data not shown), some mature βA plaques appeared (see examples in inset, Fig. 2B). Beta-sheet aggregation was further visualised by Thioflavin-S and Congo Red staining (Fig. 2C&F) thus confirming mature fibril formation, though frequently amorphous rather than fibrillary in appearance.

**Figure 3:**
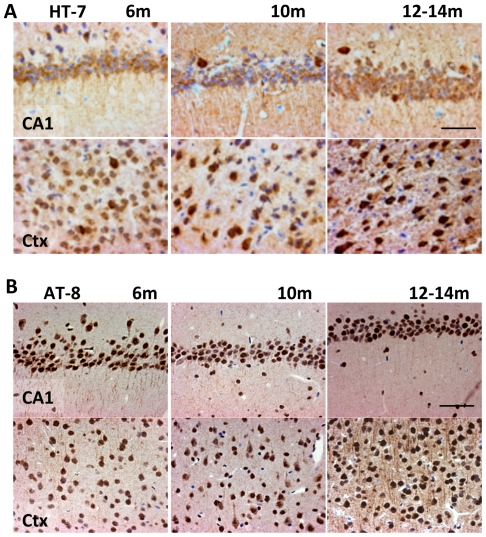
Tau histopathology in PLB1_Triple_ mice. Immunocytochemcial labelling for human tau (HT-7 antibody, **A**) as well as phospho-tau (AT-8, **B**), revealed positive staining in somata and processes in CA1 and cortical neurones from 6 months onwards in PLB1_Triple_ mice.

Quantification of plaque load in PLB1_Triple_ mice (based on 6E10 stain) at ∼6 months was sparse (∼1 plaque per 5 µM coronal brain section in cortical and hippocampal regions), this increased to 4.2+/−0.5 plaques per section at 21 months (Fig. 2E). Quantification of aggregated βA (Amorfix A^4^ assay, see Methods and Text S1 for details) also confirmed significantly enhanced levels in 12 and 20+ months old transgenic mice relative to independent C57/BL6 wild-type mice and PLB1_WT_ (P<0.001). Somewhat unexpectedly, high numbers of human tau immunoreactive (HT-7, Fig. 3A) and phospho-tau (AT8, Fig. 3B) neuronal somata emerged already in PLB1_Triple_ mice from ∼6 months onwards throughout cortex and hippocampus, labelling intensified and extended into the neuropil with age. These data confirm an age-related, predominantly intra-neuronal, neuropathology in PLB1_Triple_ mice, and suggest corresponding impairments of physiology in forebrain.

### Normal synaptic transmission, but impaired neural plasticity, in the hippocampus of PLB1_Triple_ mice

**Figure 4:**
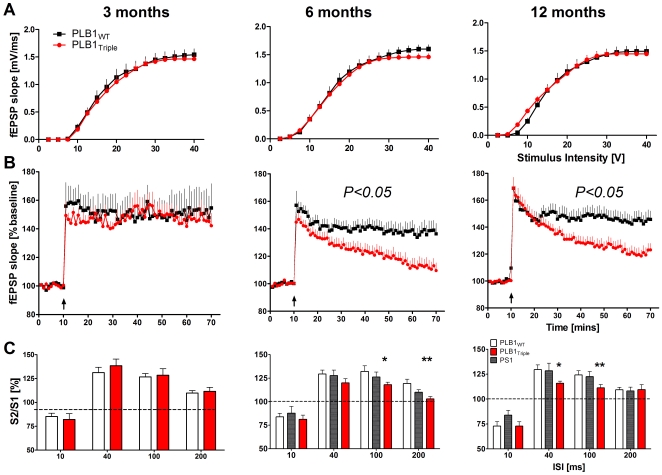
Failure in synaptic plasticity in adult PLB1 mice. Electrophysiological characterisation in the CA1 region of hippocampal slices from PLB1_Triple_ and PLB1_WT_ mice at 3, 6 and 12 months. **A:** Input-output relationships of basic synaptic transmission (fEPSP slope vs. stimulus intensity; mean+SEM) were not affected in PLB1_Triple_ mice. **B:** LTP time courses (fEPSP slope as % of baseline, +/− SEM) revealed a significant impairment at 6 and 12 months (genotype effect (*P*<0.05) and genotype x time interaction (*P*<0.001)). Arrow: time point of tetanisation. **C:** Reduced paired-pulse facilitation was uncovered in PLB1_Triple_ mice at 6 and 12 months, but not in PS1 animals and 3-month old PLB1_Triple_. Data (mean+SEM) are expressed as the slope ratio (S2/S1) vs. inter-stimulus interval (ISI). Significances are indicated as *: *P*<0.05; **: *P*<0.01; ***: *P*<0.001.

Investigations into cellular patho-physiological correlates commenced based on field EPSPs recorded in hippocampal slices [19]. Synaptic responses and plasticity in slices from mice aged 3 months did not differ between genotypes in any test protocol (Fig. 4A–C). However, while basic synaptic transmission was also intact at 6 and 12 months (*F*<1, *P*>0.05, Fig. 4A), LTP following theta-burst stimulation (Fig. 4B) decayed faster in PLB1_Triple_ mice despite normal post-tetanic potentiation. Robust potentiation of ∼140% was recorded in WT mice 60 mins post-tetanus, while the fEPSP slope in PLB1_Triple_ mice declined steadily to 113% (at 6 month) and 122% (at 12 months), respectively (genotype effect: 6 & 12 months: p<0.05; time x genotype interaction: 6 months: *F*(59,2596) = 2; *P*<0.001; 12 months: *F*(59,2478) = 4.5; *P*<0.001).

Similarly, paired-pulse facilitation (PPF, Fig. 4C), a measure of pre-synaptic short-term plasticity and transmitter release, was reduced in both PLB1_Triple_ age groups relative to WT (at 6 months: 40 ms: *P<0.05*; 100 ms: *P*<0.01; 200 ms: *P*<0.001; at 12 months: 40 ms: *P<0.05*; 100 ms: *P*<0.01). PPF in slices from age-matched homozygous PS1 transgenic mice was not affected indicating that impairments in hippocampal plasticity are endophenotypes related to hAPP/htau transgene expression. Collectively, age-dependent impairments in short- and long-term hippocampal plasticity were uncovered from 6 months of age onwards.

### Age-dependent cognitive deficits in PLB1_Triple_ mice

**Figure 5:**
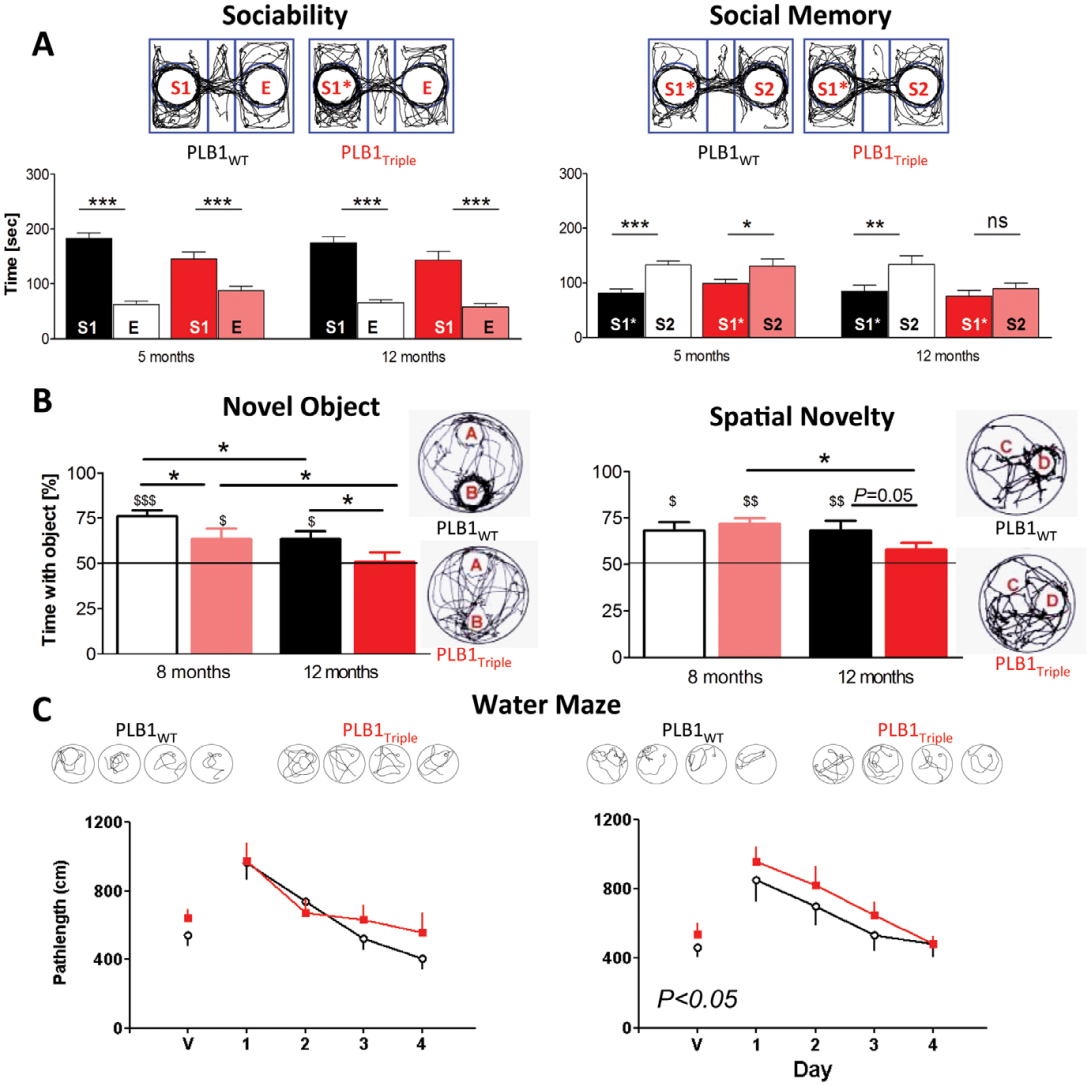
Recognition memory and learning in PLB1 mice. **A: Social recognition.** Time (in seconds) spent with an unfamiliar stranger mouse (S1) and the corresponding empty compartment (E) at 5 and 12±1 months of age. During sociability (left), all groups spent significantly more time in the vicinity of the unfamiliar mouse compared to the empty holder. During the social memory phase (right), PLB1_WT_ mice discriminated between the now more familiar first stranger (S1*) and a novel, unfamiliar stranger (S2) in both age groups. PLB1_Triple_ only discriminated between the conspecifics at 5 months. Data are represented as means+SEM; n.s.: not significant; *: *P*<0.05; **:*P*<0.01; ***: *P*<0.001, arena and representative exploration patterns are also depicted. **B: Object recognition and spatial novelty.** Time (in %+SEM) spent with a novel object (left) or relocated object (right) in PLB1_WT_ and PLB1_Triple_ mice at 8 and 12±1 months. PLB1_Triple_ performed worse than PLB1_WT_ in both age groups during object recognition and did not discriminate between novel and familiar object at 12 months of age. At this age, recognition of spatial novelty was also affected. Significances are indicated between groups (*), a comparison with chance level is also given ($:*P*<0.05; $$: *P*<0.01; $$$: *P*<0.001). Examples path trajectories are illustrated on the right. **C: Water maze learning.** Mean daily path length (+ or − SEM) to reach a visible (V) platform, followed by spatial training to a submerged platform (days 1–4). At 5 and 12 months, PLB1_Triple_ mice were able to locate the visible platform, but were significantly impaired at 12 (but not 5) months in the hidden platform task compared to PLB1_WT_ (*P*<0.05). Arena set up with representative exploration patterns for day 1–4 (swim path) are depicted.

Neuropsychological protocols in PLB1_Triple_ mice determined phenotypes congruent with the cognitive decline observed in AD patients. After confirmation of intact sensory-motor abilities (Figure S4), we explored short-term/working memory in two tasks. In line with intermediate phenotypes of AD [20], social recognition memory (expressed as time with an unfamiliar stranger vs. a more familiar stranger, Fig. 5A) was reduced in 5 months (PLB1_WT_ *P*<0.001; PLB1_Triple_ *P*<0.05) and absent in 12 months old PLB1_Triple_ (PLB1_WT_ *P*<0.01; PLB1_Triple_ *P*>0.05) while sociability towards one single stranger mouse was unaffected (all *P's* for S1 preference <0.001). This was corroborated in object recognition/displacement paradigm (Fig. 5B), in which PLB1_Triple_ mice presented with impaired recognition of a novel object at 8 months (PLB1_Triple_ vs. PLB1_WT_ *P*<0.05) and amnesia at 12 months (PLB1_Triple_ n.s. *vs.* chance level of 50%). After spatial displacement, impairment occurred only at 12 months (*P*<0.05) suggesting a delayed onset of spatial compared to object recognition memory deficits.

In agreement with these results, PLB1_Triple_ mice at 12 months of age were deficient in spatial acquisition learning as investigated in the open field water maze (Fig. 5C; genotype effect: F(1,116) = 4.5; *P*<0.05)), but not at 5 months (F(1,120) = 1.7; *P*>0.05). The acquisition deficit at 12 months was overcome by further training (see day 4; Fig. 5C). Consistently higher swim speeds were observed in PLB1_Triple_ mice in both age groups (P's<0.05), but there was no phenotype in thigmotaxis (Figure S5). These data provide evidence for some deficits in working/short-term recognition memory preceding the emergence of spatial learning impairments in PLB1_Triple_ mice, and complex deficits in all three cognitive tasks in the older age-group.

### Alterations in brain metabolism in PLB1_Triple_ mice precede the onset of cognitive deficits

**Figure 6:**
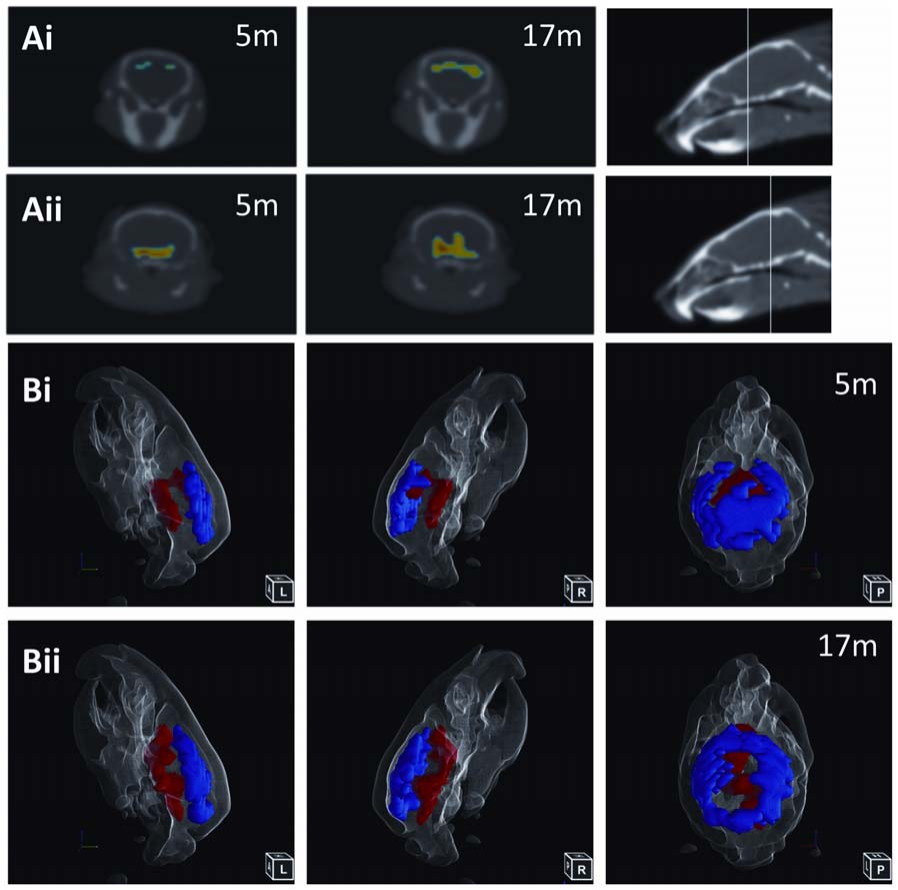
Brain metabolism: FDG PET/CT phenotype of PLB1 mice. In vivo FDG-PET images and surface renderings of PLB1Triple vs. PLB1WT animals at 5 and 17 months are depicted. Graded 2D coronal sections (**A**) show the expanding regions of decreased (**Ai**) or increased metabolism (**Aii**) (relative to whole brain activity), the location of the cross sections is indicated on the right. The full extent of metabolic group differences is illustrated at 5 (**Bi**) and 17 months (**Bii**) for lateral (L:left; R:right) and dorsal (P:posterior) views of 3D surface rendered images; areas of significant metabolic increase (red) and decrease (blue) are based on SPM at P<0.01, the reliability of clusters was also confirmed (family-wise errors, P<0.05). A surface render of a CT image provides the anatomical reference.

We next investigated whether PLB1_Triple_ mice also present with changes in brain metabolic activity via *in vivo* imaging in two age groups (5 and 17+/−1 month), with uptake of ^18^F-DG conducted under home cage conditions. After data were pooled for genotype and age, group comparison using voxel-based statistical parametric mapping (threshold set at *P*<0.01) generated 2D (Fig. 6A) and 3D (Fig. 6B) reconstructions of ^18^F-DG PET/CT images that displayed regions of significant differences between genotypes, while total FDG uptake did not differ between groups (data not shown). Region-specific metabolic activity differed between groups in both age groups, bilateral hypometabolism (depicted in blue in Fig. 6B) was already uncovered in PLB1_Triple_ cf. wild-type mice at 5 months (Fig. 6Ai and Bi), particularly in the occipital and parietal cortices, while ventral elements, encompassing the basal forebrain, striatum, thalamus and pons were metabolically hyperactive (Fig. 6Aii, and red areas in Bi). These phenotypes increased with age (see 6Ai cf. Aii, and 6Bi cf. Bii), voxel-based quantification of areas affected confirmed progression with age, i.e. regions with decreased metabolism expanded from 22.656 mm^3^ to 30.336 mm^3^ while hypermatoblism affected 24.448 mm^3^ at 5 months vs. 31.104 mm^3^ 12 months. Though a precise identification of anatomical details is difficult, structural information obtained from the Digimouse atlas and alignments with Paxino-Watson coordinates indicated that hypometabolic cortical regions extended with age to reach the rostral tip of the prefrontal region as well as the cerebellum in 17 months old PLB1_Triple_ mice (blue areas in Fig. 6B). Furthermore, areas of hypermetabolism in transgenic mice also expanded, and stretched from the posterior end of the olfactory bulb, via ventral orbital cortex, continuing throughout the basal forebrain nuclei, basal ganglia including caudate and putamen, ventral thalamus and hypothalamus, pons and continuing into the brain stem (red areas in Fig. 6B).

Therefore, PLB1_Triple_ mice displayed a dorso-ventral, bi-directional change in metabolic activity compared with their wild-type counterparts. Overall, altered glucose metabolism emerged as a an early yet progressive, transgene-induced AD-like trait in this transgenic line, offering a novel translational approach for future intervention studies.

### AD-like sleep disturbances and slowing of EEG in PLB1_Triple_ mice

**Figure 7:**
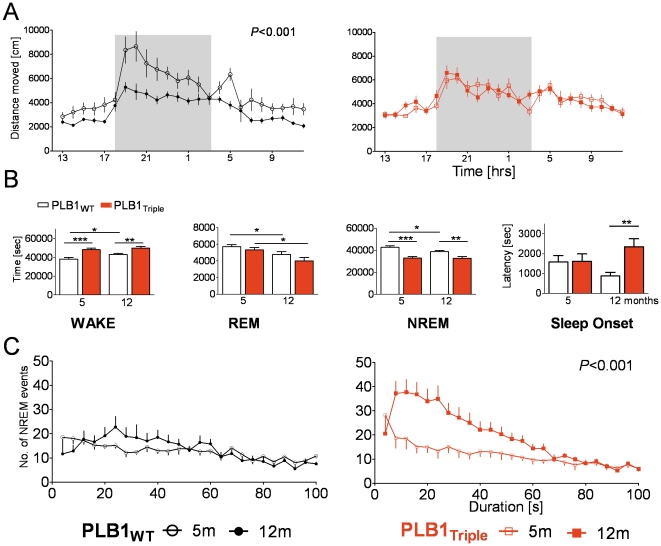
Circadian rhythm, wakefulness, and sleep architecture in PLB1_WT_ and PLB1_Triple_ animals at 5 and 12 months of age. **A:** Circadian patterns of locomotor activity (mean distance moved ± SEM, in hourly bins over 24 hrs) were intact in PLB1_Triple_ mice. Only PLB1_WT_ showed higher activity at 5 months and an age-related reduction (*P*<0.001). **B:** Time (in seconds+SEM) spent in vigilance stages wakefulness (WAKE), REM and NREM sleep, and sleep onset (latency). PLB1_Triple_ animals display increased wakefulness and corresponding reduction in NREM sleep at both ages (genotype effect: *P*'s<0.001). REM sleep was affected by age (*P*<0.01) but not genotype, and onset of sleep delayed in 12 m PLB1_Triple_ mice (*P*<0.01). **C:** A highly significant age-related increase in the number of short-duration NREM events was uncovered in PLB1_Triple_ (right) but not PLB1_WT_ mice (left). Data represent mean +/− SEM. *: *P*<0.05; **:*P*<0.01; ***: *P*<0.001.

Encoding of spatial relationships is subject to hippocampal processing during post-training sleep and wakefulness. These parameters are affected in AD patients and assumed to be mechanistically linked with the emerging memory deficits. As we have also observed respective changes in a conventional AD mouse models (e.g. [21]), we here explored alterations in the activity profile and sleep patterns of PLB1_Triple_ mice as key features of disease onset and progression (Fig. 7). Actimetry recorded in home-cage environments (Fig. 7A) confirmed intact circadian rhythms in both genotypes with heightened ambulatory activity during the dark periods, typical for nocturnal species. Relatively high activity in 5 months old PLB1_WT_ mice declined to lower levels at 12 m (*F*(1,408) = 88; *P*<0.001), reflecting a natural ageing trend. By contrast, no age-related decay in motor activity was noticed in PLB1_Triple_ mice (F<1), due to already low activity levels at 5 months. As a consequence, PLB1_Triple_ differed from WT mice in both age groups, with comparatively lower activity at 5 months, and higher activity at 12 months (*P*<0.01 and 0.001, respectively).

Quantitative EEG (qEEG) recorded using wireless microchip technology and EEG/activity-guided vigilance staging (over 24 hrs, in 4 sec bins) yielded significantly enhanced wakefulness in PLB1_Triple_ mice alongside reduced NREM sleep (Fig. 7B; 5 months: *P*'s<0.001; 12 months: *P*'s<0.01). Again, an age-dependent reduction in NREM sleep together with an increase in wakefulness was only present in PLB1_WT_ mice (effect of age: *P's*<0.05), but not in PLB1_Triples_ (effect of age: *P's*>0.05). Yet, both genotypes presented with an age-dependent decay in REM sleep (*P*'s<0.05), but there was no transgene-specific phenotype. Latency to first sleep occurrence was also prolonged in PLB1_Triple_ mice at 12 months (*P*<0.01) compared with age-matched PLB1_WT_ mice, but no difference was present at 5 months. Analysis of the number of NREM events in relation to their length (Fig. 7C) revealed the occurrence of short bouts of NREM episodes in the different cohorts. NREM composition was not different at 5 months but short events significantly increased at 12 months in PLB1_Triple_ mice (genotype effect: *F*(1,350) = 54; *P*<0.001; genotype x bout duration: *F*(24,350) = 3.3; *P*'s<0.001). It thus appears that PLB1_Triple_ mice show an early vigilance and activity phenotype that comprises increased wakefulness amidst reduced motor activity, which further progresses to yield a delay in sleep onset and NREM fragmentation at 12 months of age.

**Figure 8:**
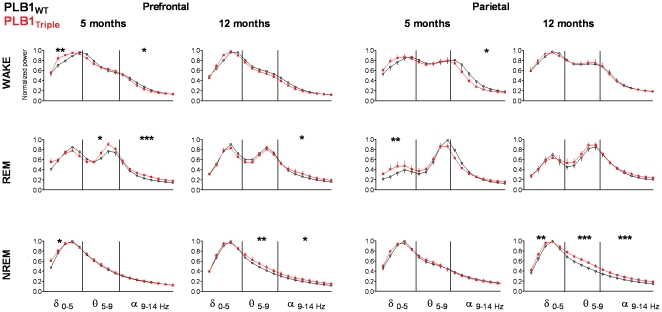
Vigilance-stage specific EEG power spectra in PLB1_WT_ and PLB1_Triple_ animals at 5 and 12 months of age. Normalised power spectra (delta - alpha bands) from specified vigilance stages are depicted for recording sites above the prefrontal cortex (left) and parietal cortex (right), significances between genotypes are indicated per frequency band. A shift towards lower frequencies and thus a rise in delta power was observed at 5 months, while NREM EEGs showed global genotype- and age-related changes at 12 months. *: *P*<0.05; **:*P*<0.01; ***: *P*<0.001.

Shifts in qEEG spectral power are recognised early indicators for AD [22], [23]. In agreement with this endophenotype, an increase in delta power was observed in 5 months old PLB1_Triple_ mice during wakefulness (*F*(1,90) = 7; *P*<0.01) and NREM (*F*(1,90) = 2.9; *P*<0.05) in the prefrontal EEG power spectrum (Fig. 8); this was paralleled by higher delta power during REM sleep from parietal recordings (*F*(1,80) = 7; *P*<0.01). A reduction in alpha power was consistently uncovered at both recording positions (*F*'s>5; *P*'s<0.05) during wakefulness. Interestingly, a generalised widening of normalised EEG spectra during NREM emerged in PLB1_Triple_ mice at 12 months (genotype effect prefrontal: theta band *P*<0.01, alpha band *P*<0.05; parietal: delta band *P*<0.01, theta and alpha bands *P's*<0.001), confirming this as a progressive biomarker accompanying sleep fragmentation, while a prefrontal increase in delta power in awake animals appears indicative of early pathology.

## Discussion

We here present a phenotypic analysis of a novel transgenic AD mouse based on targeted knock-in of mutated hAPP and hTau transgenes. The rational of this approach was a) to avoid positional effects and mutations of the endogenous mouse genome, a natural by-product of mice generated via pronuclear injections [5]; b) to generate a genetically stable AD model based on single copy gene insertion that can be reliably identified over future generations; c) employ a neurone- and forebrain-specific regulatory element to direct transgene expression towards AD-relevant brain structures and avoid expression in brainstem and cerebellum which may cause confounding motor phenotypes; and d) to generate a model that permits the generation of genetically directly comparable single hAPP and hTau transgenic mice after deletion of either gene. Not unexpected given the genetic design, PLB1_Triple_ mice displayed low levels of extracellular protein accumulation and plaque formation was sparse. Nevertheless, intra-neuronal APP/βA and tau pathologies were reliably identified and successful transgene processing confirmed further in a parallel study, where the same genetic constructs were delivered to different neuronal preparations by viral transduction [24]. This approach provided an additional proof of principle for transgene processing and confirmed mechanisms of neurotoxicity, as secretase and aggregation inhibitors successfully ameliorated toxicity.

In the mouse model described here, expression of single gene constructs created a patho-physiology reminiscent of early, prodromal AD consisting of intraneuronal build-up of APP/βA and tau that appear prior to plaque and tangle deposition. These constitute early markers of neurotoxicity that lead to synaptic failure and functional loss [15], [25], [26]. The emergence of intraneuronal amyloid correlated with deficits in synaptic plasticity and cognition in previous studies [3], [27], but intracellular βA oligomeric peptides are not efficiently detected by ELISAs [28], [29]. Though more aggressive gene expression and protein build-up achieved in previous transgenic mice are convenient for quantification, this approach makes it difficult to dissect early phenotypes such as subtle [3]cognitive or physiological deterioration [1], [5], [30], [31]. Detection of βA oligomers in the PLB1_Triple_ mice required the use of an ultra-sensitive assay specifically designed to detect low concentrations of aggregated βA. The presence of low but measurable levels of aggregated βA correlate with the physiological and behavioural deficits which have been characterized in these mice. Low expression of mutated transgenes in PLB1_Triple_ mice offers the advantage of a prolonged prodromal phase, leading to moderate cognitive and physiological impairments in rodents at ∼12 months. Correspondingly, expression of hAPP with the E693Δ mutation in transgenic mice generated by pronuclear injection caused exclusively intracellular oligomeric βA build-up [15]. Despite a lack of extracellular βA aggregates, hippocampal plasticity and memory declined age-dependently and phenotypes were similar to the deficits observed here in LTP and spatial learning in PLB1_Triple_ mice. However, overall impairments in rodent models expressing intraneuronal βA remain mild [32], possibly due to the lack of tau pathology. Co-expression of mutated βA and tau in our PLB1_Triple_ mice progressively affected synaptic plasticity and ultimately cognition. While its origins in PLB1_Triple_ remain to be fully resolved, neuronal failure was also induced in mice with selective expression of the intracellular APP domain (AICD), without increasing βA itself [33], or in rTg4510 mice expressing soluble tau [34]. Taken together, evidence is now clearly accumulating for the relevance of early-stage pathologies, modeled by regulated and specific gene expression, with a focus on soluble rather than fibrillary histo-pathologies. With regards to the early tau expression detected here with an unusual pattern (pronounced HT7 and AT8 stain in nearly all hippocampal and cortical principle neurons from 6 months onwards), our model clearly differs from previous lines (e.g. [35]), thus calling for further analyses of its association with specific cellular compartments and the role of specific phosphorylation sites.

A second objective of our study was the development of sensitive translational procedures based on *in vivo* physiological and imaging parameters that are coincident with the emergence of intraneural amyloid/tau and precede a cognitive symptomatic state. Effective detection of reliable, early translational biomarkers is a major obstacle not only in research but also in the clinic, where diagnosis of conversion from MCI toward early dementia is crucial to identify autonomy impairments in domestic, social, or professional aspects of life. Deficits in social and facial recognition are typically absent in MCI and appear on conversion to early AD [36]. We here report a corresponding phenotype in 12-month old PLB1_Triple_ mice, which may be related to anomalies in cholinergic processing [20]. Similarly, working memory, the capacity to maintain and manipulate information during short time periods, is often maintained in MCI (see [37] for references) but deteriorates on conversion to early AD, in agreement with recognition and spatial memory deficits emerging in older PLB1_Triple_ mice. Early, but purely experimental, parameters affected comprised hippocampal synaptic plasticity, impaired at 6 months when APP/βA and tau were first detected. Potentially clinically relevant, early translational biomarkers consisted of metabolic (FDG) changes that affect dorsal cortices and basal forebrain structures, as well as EEG spectral power and sleep irregularities, mapping both aging and transgene-related disease progression [38]–[40]]. Although the function of different sleep stages in memory processes is controversial [41], [22], NREM sleep seems critical for the consolidation of declarative (spatial) memory, and REM for non-declarative memory [42]. Slowing of cortical EEG, reduced NREM sleep and fragmentation in PLB1_Triple_ mice may explain deficits in memory formation in a manner reminiscent of sleep deprivation, which affects mechanisms required for learning-induced long-term plasticity, and consistent with an early LTP phenotype. Again, contributions of βA and tau remain the subject of future work, but a similar endophenotype may underlie matching symptoms in patients with early AD [43]. Such a comprehensive phenotype has not been found in plaque bearing APP/PS1 mice [44], [45], implicating a potential role of intraneuronal tau protein in sleep regulation and circadian function.

The causes and consequences of an overall slowing of EEG likely reflect failing synchronisation and connectivity between brain regions [46]–[49]. Reduced alpha and heightened delta/theta power during wakefulness and more prominently during REM sleep are reliable endophenotypes of AD [22], [23], [39]. It is conceivable that missing intra- or inter-frequency phase synchrony would recruit fewer neuronal assemblies for large scale integration [50], [51] and explain working/short-term memory deficits [52] observed in 12 months old PLB1_Triple_ mice.

A reliable metabolic PET phenotype in AD models has so far also remained elusive [53], [54]. FDG-PET studies conducted in Tg2576 mice identified areas of hypermetabolism in young-adult (7-month old) transgenic mice cf. wild-types, but failed to uncover differences in aged mice [54]. These data suggest that extracellular amyloid accumulation does not correlate with metabolic changes in AD relevant brain areas, and both prodromal and early-AD-like metabolic endophenotypes may be uniquely mimicked in the PLB1_Triple_ model. Some metabolic (FDG) changes based on autoradiography have been reported in the 3xTG mouse [55], but resembled a more global reduction in FDG uptake with little AD-relevant brain region specificity. The use of *in vivo* FDG-PET in preclinical studies is particularly interesting as a diagnostic tool and for repeated longitudinal studies. Although amyloid or tau-specific PET ligands would constitute major progress for research and diagnosis, experimental work at present still seeks to establish an agreed procedural gold standard for meaningful comparisons with clinical imaging. Results from PET studies have to be interpreted on the basis of the applied registration and normalisation procedures, and imaging results often reveal changes in metabolism that are not immediately intuitive [56]. Therefore, the apparent hypermetabolism in subcortical structures of PLB1_Triple_ mice may reflect changes relative to whole brain activity consequent to disease progression or age, phenotypes may occur as a result of inflammation and metabolic activity of glia [57] or display compensatory neuronal activity. Interestingly, regions of hypometabolism overlap with areas of transgene expression and physiological (EEG, LTP) changes. This is an intriguing finding given the different time-scale of recording (30 mins for PET, 1 hr of LTP, 24 hours for EEG), but compatible with the notion that symptoms represent early phenotypes of a progressively deteriorating neural network.

In summary, the less aggressive but genetically stable nature of PLB1_Triple_ mice has created a phenotype of prodromal AD/MCI that has lower levels of APP/βA and tau expression compared to conventional over-expression models, but allowed us to establish sensitive translational procedures (microPET/CT imaging and wireless EEG recording techniques), alongside standard experimental endpoints. Such approaches have the potential to focus translational research and treatment efforts on early stage AD, the optimal window for intervention.

## Materials and Methods

### Transgenic mice

All mice were housed and tested in accordance with European (FELASA) and UK Home Office regulations, experiments were approved by the university's Ethics Board and carried out in accordance with the Animal (Scientific Procedures) Act 1986 (PPL 60/4085). Mice were kept in a holding room with a 12 hour light/dark cycle, temperature was maintained at 23±2°C and 40–60% relative humidity. Animals were allowed free access to food (standard rodent chow) and water. Independent, naive cohorts of mixed gender were used for a range of *in vivo* experimental procedures between 3 and 21 months of age (group details and n's as specified below), unless stated otherwise. After in vivo experimentation, issue was harvested for histological analyses.

PLB1 mice were generated by targeted knock-in of a human APP-Tau cDNA construct (see Fig. 1A: hAPP: isoform 770 containing Swedish and London mutations; htau: isoform 2N4R with P301L and R406W mutations) under the control of the mouse CaMKIIα promoter [58] cloned into the HPRT™ targeting vector [59], conducted by genOway, France). To stabilise expression, an artificial intron derived from a pNN265 vector was fused to the regulatory element and an IRES sequence inserted between transgenes. Additional LoxP and FRT sites flanking hAPP and hTau cDNAs, respectively, will enable selective deletion of either gene in future studies. Gene targeting was performed in E14Tg2a ES cells subsequently injected into C57BL6/J blastocysts. APP-Tau expressing animals (PLB1_Double_) were crossed with hPS1 (A246E) transgenic mice [60] to generate PLB1_Triple_ animals (hAPP/hTau/hPS1) and transgenic status confirmed using PCR and Southern Blot (Figure S1). The *Hprt* locus is located on the X chromosome, thus mating of heterozygous PLB1_Triple_ females with transgenic (hemizygous) males generated either heterozygous or homozygous females and hemizygous or WT males. Additional WT controls were derived from parallel matings between heterozygous PLB1_Triple_ females with male PLB1_WT_. For clarity, all animals from PLB1 crossings that do not carry any transgenes are referred to as PLB1_WT_.

Genotyping for transgenic cDNA insertion PCR amplification used forward primer GW496: 5′ -ACA ATT GCC TGT GAA TCA AGT TCT AGA TCT GG - 3′ and reverse primer GW497: 5′ -TTC GTC CAG ATC ATC CTG ATC GAC AAG AC - 3′. In addition, *Hprt* wild type (WT) primer pairs 5′ -TGT CCT TAG AAAACA CAT ATC CAG GGT TTA GG -3′ and 5′ -CTG GCT TAA AGA CAA CAT CTG GGA GAA AAA -3′, enabled the distinction between hetero- and homozygous animals.

*For RNA extraction and quantitative real-time PCR* (6 months: n = 5 for each genotype; 12 months: n = 3 (homozygous PLB1_Triples_) or n = 4 (all other groups)) brain samples (4 mm^3^) were stored in ‘RNA Later’ solution until extraction with RNeasy Lipid Tissue Mini Kit (manufacturer's instructions). Only integrity-controlled RNA (Agilent 2100 Bioanalyzer, Cheshire, UK, RIN-score >7) was used for cDNA synthesis with Transcriptor High Fidelity Reverse transcriptase kit (Roche, Burgess Hill, UK) and gene expression confirmed with Bio Rad MiniOpticon Real-Time PCR Detection System using iQ SYBR Green Supermix (BioRad, Hemel Hempstead, UK) in a final volume of 20 µl. A 100 ng cDNA equivalent was run (in triplicates) per sample with 3.2 µM each with transgene (APP and tau) and GAPDH (housekeeping gene) specific oligomer primers. Quantification was conducted against standard serial dilutions of plasmids, and copy numbers normalised vs. GAPDH (Opticon Monitor™ Software, BioRad, Hemel Hempstead, UK).

*Tissues harvesting and histology* (PLB1_WT_ n = 3–4, PLB1_Triple_ and n = 4–6 per age group) followed procedures described in [21]. Briefly, brain sections were stained with amyloid antibodies 6E10 (1∶200, Cambridge Bioscience) or DE2B4 (1∶200, Abcam, Cambridge, UK), HT-7 (1∶200, Autogen-Bioclear, Wiltshire, UK) for human-specific tau and AT-8 (1∶25, Autogen-Bioclear) for phospho-tau. Plaque load in PLB1_Triple_ was quantified by manual counting based on the 6E10 staining (n = 14). Beta-sheet protein aggregation was further detected using Thioflavin-S (1%, Sigma-Aldrich) and Congo Red (0.5%, Sigma). Images were taken with a digital camera (Axocam, Carl Zeiss; Hertfordshire, UK) mounted on a Zeiss microscope (Axioskop 2 Plus).

*For detection of aggregated βA* an ultra-sensitive assay was employed in forebrain samples from 12 months old C57/BL6 WT (n = 5), as well as in transgenic mice (12 months: n = 11; 20+months: n = 9) and PLB1_WT_ (n = 12) animals (A4 assay, Amorfix, Ontario, Canada). Briefly, a proprietary sample enrichment protocol was used to isolate oligomeric βA (see Text S1 for further details). Following enrichment, samples were eluted and disaggregated to allow detection of βA [61] based on an immunoassay using europium-fluorescent beads coupled to the mouse monoclonal 4G10 antibody (N-terminal βA, aa 1–17) and magnetic beads coupled to the antibodies 1F8 (C-terminal βA, aa 3–40) and 2H12 (C-terminal βA, aa 3–42). The intensity of the europium fluorescent signal was measured using time resolved fluorescence (TRF) on each sample in triplicate and was taken as being directly proportional to the concentration of aggregated βA within the sample. The limit of detection using this technique is 50 fg of protein per well.

*In vitro hippocampal slice electrophysiology* (4–6 animals per each age group and genotype, number of slices listed below) followed our previous protocol (e.g. [19] with minor modifications. Briefly, hippocampi were dissected in ice-cold sucrose aCSF (composition in mM): 249.2 sucrose, 1.5 KCl, 1.3 MgSO_4_, 1.5 KH_2_PO_4_, 2.89 MgCl_2_.6H_2_O, 0.96 CaCl_2_, 25 NaHCO_3_ and 10 glucose (pH 7.4, continuously gassed with 95% O_2_/5% CO_2_). Slices (400 µm) were stored in pre-warmed, oxygenated aCSF (composition in mM: 129.5 NaCl, 1.5 KCl, 1.3 MgSO_4_, 2.5 CaCl_2_, 1.5 KH_2_PO_4_, 25 NaHCO_3_ and 10 glucose; 32°C) for at least 1 h before experimentation commenced.

Field excitatory postsynaptic potentials (fEPSPs) were recorded in area CA1, evoked by stimulation of the Schaffer collateral fibres. Stepwise increases of stimulus intensity until saturation revealed input/output curves of basic synaptic transmission of fEPSP slopes (3 months: PLB1_WT_ n = 25, PLB1_Triple_ n = 24; 6 months: PLB1_WT_ n = 16, PLB1_Triple_ n = 13, 12 months: PLB1_WT_ n = 26, PLB1_Triple_ n = 12).

LTP (3 months: PLB1_WT_ n = 8, PLB1_Triple_ n = 11; 6 months: PLB1_WT_ n = 23, PLB1_Triple_ n = 13, 12 months: PLB1_WT_ n = 16, PLB1_Triple_ n = 12) was induced by a theta-burst stimulation (5 Hz, 5 bursts of 4 stimuli (100 Hz), inter-burst interval of 200 msec for 1 second, stimulus intensity: 50% of maximum) and recorded up to 60 min post-tetanus.

A paired-pulse protocol with inter-stimulus intervals (ISIs, stimulus intensity: 50% of maximum) of 10, 40, 100 and 200 ms investigated changes in presynaptic transmitter release and short-term plasticity (3 months: PLB1_WT_ n = 16, PLB1_Triple_ n = 11; 6 months: PLB1_WT_ n = 10, PLB1_Triple_ n = 11, PS1 n = 10; 12 months: PLB1_WT_ n = 16, PLB1_Triple_ n = 11, PS1 n = 10).

*Social Recognition* (5–6 months: PLB1_WT_ n = 41; PLB1_Triple_ n = 41; 12–13 months: PLB1_WT_ n = 24; PLB1_Triple_ n = 24) was conducted as detailed before in [20]. The apparatus consisted of a three-chambered box, both side-chambers contained a cylinder for confinement of a stranger mouse. Test sessions consisted of habituation, followed by sociability (presentation of one unfamiliar stranger, S1) and a social memory phase (with a novel, unfamiliar stranger (S2) and the now familiar stranger (S1*)), 10 min each, inter-trial interval: 5 mins. Path trajectories in a target area indexing direct social contacts (4 cm) were video-recorded and stored online (Ethovision; Noldus IT, Netherlands). Social interactions were quantified as contact time with S1 cf. the corresponding empty compartment (sociability), or with S2 cf. S1 (social memory). Animals that showed little exploration (total of <10 sec in target zone) were excluded (n = 2), two animals were excluded as outliers (>2SD from mean).

*Object recognition* (8 months: PLB1_WT_ n = 15; PLB1_Triple_ n = 14; 12 months: PLB1_WT_ n = 11; PLB1_Triple_ n = 14) followed a protocol described in [62] with minor modifications. Movement (path length) in the apparatus (Perspex cylinder, 50 cm diameter; 50 cm wall height) was tracked (Ethovision Pro 3.1) during 1) Habituation (2 days, 2 trials, 5 min, ITI: 2 min; trial 1: empty arena; trial 2: single object) 2) Object novelty: presentation of two identical objects A (sample phase) followed by one identical (A) and one novel (B) object (test phase) and 3) Spatial novelty: two novel objects C and D (sample phase); displacement of one object (test phase). Object exploration was recorded as % time within the object perimeter (4 cm). Exclusion criteria: object bias during the sample phase (n = 2); no exploration of objects (n = 2); data outliers (n = 2).

*Open Field Water maze* testing (5–6 months: PLB1_WT_ n = 16, PLB1_Triple_ n = 16; 12–13 months: PLB1_WT_ n =  15, PLB1_Triple_ n = 16) was modified from previous protocols [63]. Animals were allocated target platform locations (Ugo Basile, rising platforms) in the centre of one quadrant (counterbalanced design) of a circular water-filled pool (150 cm diameter, 50 cm depth) and pseudo-randomly released (maximum swim time: 90 s) from 4 cardinal points (N,E,S,W). They received 4 trials each day for 5 consecutive days (inter-trial interval 30 min) and swim paths were tracked by video software (Any-Maze; Ugo Basile) and video-stored (mpeg files). Day 1: Visible platform pre-training (with curtains). Days 2-5: Submerged constant platform location, no curtains. The following parameters were extracted: i) path length to platform; ii) swim speed; and iii) thigmotaxis (wall hugging). One mouse in each genotype was excluded for abnormally long swim-paths (>2SD from group mean).

*Circadian activity, sleep & electroencephalogram (EEG)* assessments were conducted in a longitudinal design at 5 and 12 months of age and followed protocols and procedures outlined in detail previously [21] (PLB1_WT_ n = 10, PLB1_Triple_ n = 11). Briefly, circadian rhythms were recorded in PhenoTyper home cages (Noldus IT, Netherlands) and activity (distance moved) was sampled (Ethovision, 3.1; Noldus, NL) and averaged into 1 hour bins over a 24 hr cycle (group means +/−SEM) using in-house ‘Mnimi’ software. After 2 days of habituation, EEG recordings commenced.

Cranial electrode implants were stereotaxically positioned above the left and right parietal cortex/dorsal hippocampus (2 mm posterior to Bregma/1.5 mm lateral to midline), the right medial prefrontal cortex (2 mm anterior of Bregma, 0.2 mm lateral to midline), and reference/ground electrodes placed at neutral locations as described previously. Post surgery recovery lasted >7 days. EEG was continuously recorded for 24 hrs with a wireless EEG device (Neurologger, New Behaviour, Switzerland), that contains a built-in accelerometer (movement) and 4 channels for recordings (200 Hz sampling, low pass filtered (1–50 Hz)).

Data were downloaded, converted using EEG_Process (Matlab 7, The MathWorks Inc., Natick, USA), and imported into SleepSign (Kissei Comtec Co. Ltd, Nagano, Japan) for vigilance staging (wakefulness (wake), REM and NREM sleep) and extrapolation of power spectra from 4 sec epochs. Fast Fourier Transform (FFT) was calculated with a resolution of 0.77 Hz, Hamming window smoothed, and averaged. The spectral bands of delta (0.5–5 Hz), theta (5–9 Hz), alpha (9–14 Hz), and beta (14–20 Hz) and gamma (20–50 Hz) were normalised relative to the absolute maximum power over all frequency bands. Spectral EEG characteristics of each epoch were pooled for NREM, REM and wakefulness, based on accelerometer activity and parietal/hippocampal spectral power (delta and theta power). Time spent in different vigilance stages was calculated for the 24 hr recording cycle and distribution of stages analysed (number of events vs. duration) as an index of fragmentation.

*PET Imaging* (6 months: PLB1_WT_ n = 16; PLB1_Triple_ n = 7; 17 months: PLB1_WT_ n = 12, PLB1_Triple_ n = 13) commenced in animals fasted overnight and injected intraperitoneally with ^18^F-FDG (range: 9.84–17.32 MBq in 0.33–0.5 ml) while conscious. Uptake (in darkness, 45 mins) occurred on a heating pad (35°C). For imaging, animals were anesthetized (100 mg/ml Vetalar®)/medetomidine and 1 mg/ml Domitor®) and scanned in supine position. CT and PET data were collected using a Suinsa ARGUS dual-ring scanner. The CT was obtained first (at 40 kV and 140 µA beam current) followed by a 40 minute list-mode PET acquisition (250–700 keV). 3-Dimensional (3D) sinograms were reduced to 2 dimensions by Fourier rebinning and reconstructed using two-dimensional ordered subsets expectation maximization reconstruction algorithms, including corrections for random coincidence counts, attenuation and photon scatter.

Registration processing to a standard template for voxel-based analysis were carried out using the Pmod suite (Pmod Technologies, CH), data were manually aligned with the CT and registered to the Digimouse atlas [64] inclusive non-linear warping using the Brain Norm II algorithm.

Voxel (volume: 0.064 mm^3^) normalisation was performed cf. whole brain. Statistical Parametric Mapping (SPM, Functional Imaging Lab, London, UK) determined genotype and age effects (at *P*<0.01 level). Uncorrected SPMs (at p<0.01) produced clusters of statistically significant voxels which were next subjected to a second-level statistical analysis (family-wise errors, *P*<0.05). Corresponding regions of reliable metabolic increase (red) and decrease (blue) were mapped onto a CT image for display.

### Statistics

Unless indicated otherwise, statistical analyses were performed with Prism (V.5, GraphPad, USA) using parametric or non-parametric analysis of variance (one- or two-way as appropriate) followed by suitable post-tests for selected data pairs. *P*<0.05 was considered reliable.

## Supporting Information

Figure S1 **Genotyping and confirmation of transgene knock-in. A: PCR of the F1 generation.** The genotypes of the 36 pups derived from the F1 breeding were tested by PCR using primer combinations that detect the targeted *Hprt* allele. 5 of 36 animals tested were identified as being heterozygous for the *Hprt* knock-in. DNA from the targeted ES clone #5B10 was used as a positive control. PCR without template served as a negative control. M: 1 kb DNA-Ladder (NEB). **B: Southern Blot analysis of the F1 generation.** The genomic DNA of the 2 tested F1 mice (#17763, #17764) were compared with wild-type DNA (129ES, BL6). The NheI digested DNAs were blotted on nylon membrane and hybridised with a 5′ probe to validate the zygocity of the *Hprt* gene mutation in these animals.
(PDF)

Figure S2 **Body weight of PLB1_Triple_ and PLB1_WT_ animals at 5–12 months of age.** An overall effect of gender was observed in both genotypes (***: P<0.001), and a significant effect of age was noticed in both males (P<0.01) and females (P<0.05). Data are expressed as means +/− SEM; n's at 5,7,9 and 12 months were for females: 34,18,65,32 and males:32,19,56,52, respectively.
(PDF)

Figure S3 **Examples of negative and positive controls for immunoctychemistry.** Sections from PLB1_WT_ (14 months) and from an APP/PS1 over-expressing mouse (12 months (*13*)) are shown for APP antibody DE2B4 and tau antibody HT-7. Scale bar: 50 µm.
(PDF)

Figure S4 **Intact motor performance in PLB1 mice at 10 and 16 months of age.** **A:** Balance Beam: Latency to reach the end of a 50 cm long beam of different size (square, 5, 11 and 28 mm). Means for all groups are shown (SEM omitted for clarity). **B:** Rotarod: Active performance sustained on a rotating rod (means plus SEM). There was no genotype difference between the tested cohorts, but an age effect was observed in both paradigms. *** indicates a highly significant age effect (*P*<0.01) in both genotypes.
(PDF)

Figure S5 **Thigmotaxis and swim speed during the water maze task.** **A:** Even though PLB1_Triple_ spent significantly more time in the thigmotaxis zone on day 1 (visible platform training, V), there was no overall genotype effect, and PLB1_Triple_ and PLB1_WT_ mice equally reduced the time spent in the thigmotaxis zone during spatial learning (overall *P*>0.05). **B:** PLB1_Triple_ mice presented with significantly enhanced velocity during spatial learning. This was particularly pronounced at 5 m of age (left, *P*<0.001) but also observed at 12 m (right, *P*<0.01). Accordingly, pathlength and not latency data are presented in the main manuscript.
(TIF)

Text S1 **Details of the Amorfix A^4^ procedure.**
(DOCX)
