# Supplementary figures and images for: Abnormal Cognition, Sleep, EEG and Brain Metabolism in a Novel Knock-In Alzheimer Mouse, PLB1
Source: PLoS One. 2011 Nov 11;6(11):e27068. doi: 10.1371/journal.pone.0027068 (PMC3214038; doi:10.1371/journal.pone.0027068)

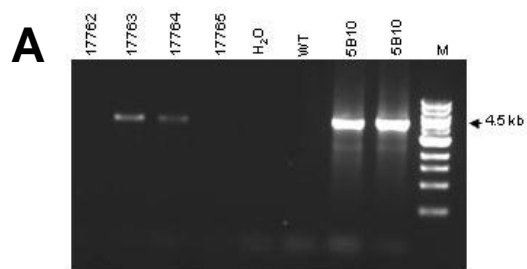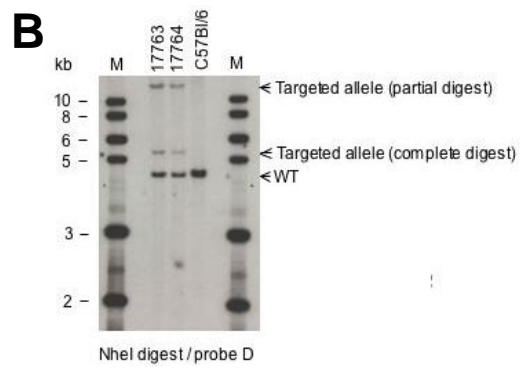

Supplement: Figure S1 — Genotyping and confirmation of transgene knock-in. A: PCR of the F1 generation. The genotypes of the 36 pups derived from the F1 breeding were tested by PCR using primer combinations that detect the targeted Hprt allele. 5 of 36 animals tested were identified as being heterozygous for the Hprt knock-in. DNA from the targeted ES clone #5B10 was used as a positive control. PCR without template served as a negative control. M: 1 kb DNA-Ladder (NEB). B: Southern Blot analysis of the F1 generation. The genomic DNA of the 2 tested F1 mice (#17763, #17764) were compared with wild-type DNA (129ES, BL6). The NheI digested DNAs were blotted on nylon membrane and hybridised with a 5′ probe to validate the zygocity of the Hprt gene mutation in these animals. (PDF) [file pone.0027068.s001.pdf]

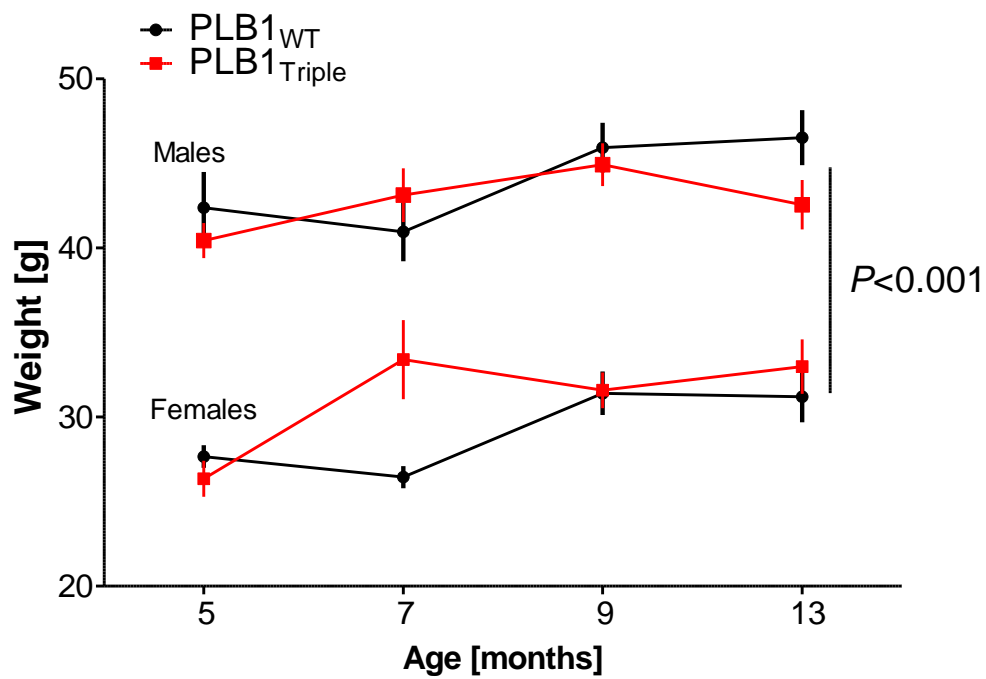

Supplement: Figure S2 — Body weight of PLB1Triple and PLB1WT animals at 5–12 months of age. An overall effect of gender was observed in both genotypes (***: P<0.001), and a significant effect of age was noticed in both males (P<0.01) and females (P<0.05). Data are expressed as means +/− SEM; n's at 5,7,9 and 12 months were for females: 34,18,65,32 and males:32,19,56,52, respectively. (PDF) [file pone.0027068.s002.pdf]

WT, 14m

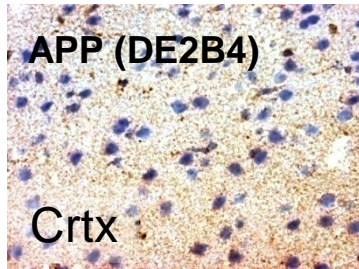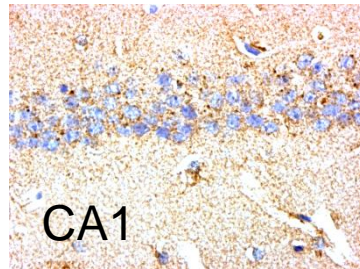

APP/PS1, 12 m

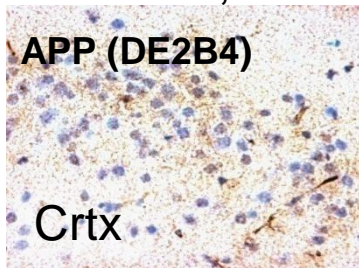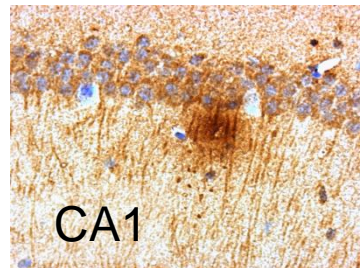

WT, 14m

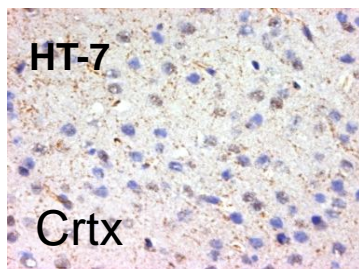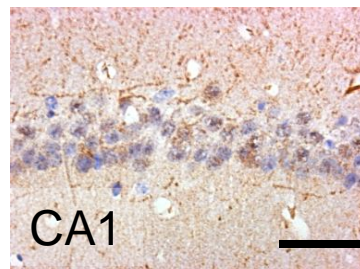

Supplement: Figure S3 — Examples of negative and positive controls for immunoctychemistry. Sections from PLB1WT (14 months) and from an APP/PS1 over-expressing mouse (12 months (13)) are shown for APP antibody DE2B4 and tau antibody HT-7. Scale bar: 50 µm. (PDF) [file pone.0027068.s003.pdf]

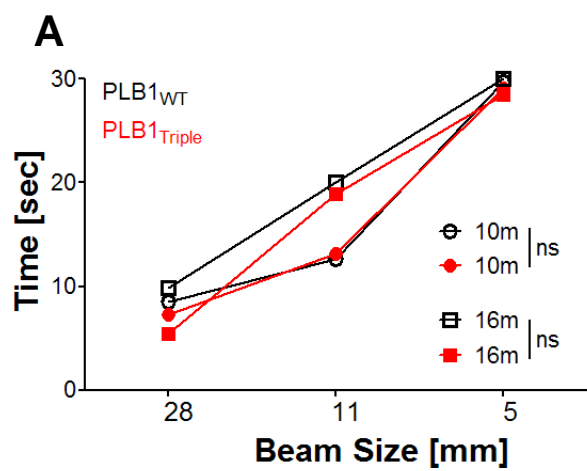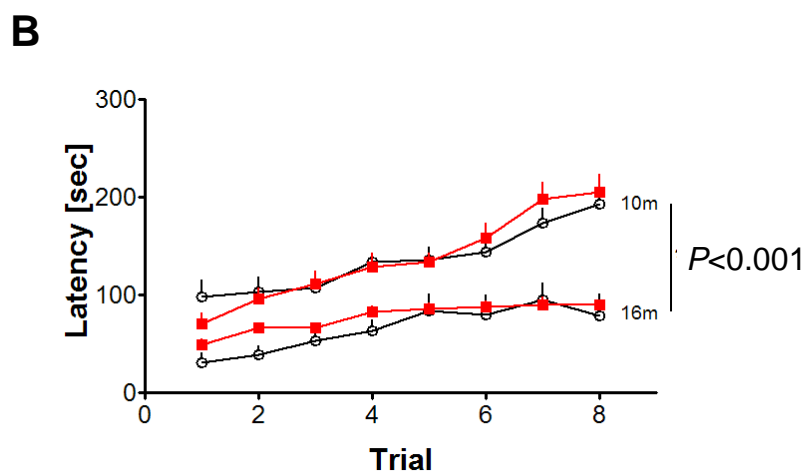

Supplement: Figure S4 — Intact motor performance in PLB1 mice at 10 and 16 months of age. A: Balance Beam: Latency to reach the end of a 50 cm long beam of different size (square, 5, 11 and 28 mm). Means for all groups are shown (SEM omitted for clarity). B: Rotarod: Active performance sustained on a rotating rod (means plus SEM). There was no genotype difference between the tested cohorts, but an age effect was observed in both paradigms. *** indicates a highly significant age effect (P<0.01) in both genotypes. (PDF) [file pone.0027068.s004.pdf]
