# Supplementary material for: Abnormal Cognition, Sleep, EEG and Brain Metabolism in a Novel Knock-In Alzheimer Mouse, PLB1
Source: PLoS One. 2011 Nov 11;6(11):e27068. doi: 10.1371/journal.pone.0027068 (PMC3214038; doi:10.1371/journal.pone.0027068)
Supplement: Text S1 — Details of the Amorfix A4 procedure. (DOCX) [file pone.0027068.s006.docx]

**Background and details of the A^4^ Assay:**

For detection of aggregated βA an ultra-sensitive, commercially available assay for the detection of aggregated species of βA (Amorfix A^4^, Amorfix Life Sciences, Ontario, Canada) was employed to quantify levels of aggregated βA in 12 months old C57/BL6 WT (n=5), as well as in transgenic 12 months: n=11; 20+months: n=9) and PLB1_WT_ (n=12) animals.

All samples were tested in a blinded manner with the identity of the samples provided only after all data was obtained and analyzed.

1. Sample Preparation:
   1. Preparation of brain homogenates:
      1. A 10% (w/v) brain homogenate was prepared from each brain (in PBS/2% NP-40 containing protease inhibitors)
   2. Brain lysates were diluted in assay buffer to provide a signal within the linear range of the immunoassay.
   3. Using a proprietary sample enrichment protocol the aggregated Aβ was isolated from each sample. Samples were loaded onto the enrichment matrix in a 96-well plate format. Following loading, the enrichment matrix was washed twice.
   4. Following enrichment, each sample was eluted and disaggregated to allow for detection using the Amorfix Aβ immunoassay.
2. Analysis: Aβ Immunoassay:
   1. The signal from each sample was detected using an immunoassay that makes use of the antibodies 4G10 (N-terminal), 1F8 (C-terminal for A 1-40) and 2H12 (C-terminal for Aβ 1-42).
      1. 4G10 was coupled to Europium beads to provide a fluorescent read-out signal.
      2. 2H12 and 1F8 were coupled to magnetic beads in order to isolate the immune complex.
      3. Samples were incubated with 4G10-coupled beads at 37^o^C with shaking.
      4. Magnetic beads coupled to 1F8 and 2H12 were added to the samples, and incubated at 37^o^C with shaking.
      5. Following all incubations, the samples were washed and placed on a magnet to isolate the immune complex.
      6. The europium fluorescence intensity was measured using time resolved fluorescence (TRF) on each sample in triplicate and is directly proportional to the concentration of βA within the sample. The limit of detection using this technique is 50 fg of protein per well.
